# P301S Mutant Human Tau Transgenic Mice Manifest Early Symptoms of Human Tauopathies with Dementia and Altered Sensorimotor Gating

**DOI:** 10.1371/journal.pone.0021050

**Published:** 2011-06-15

**Authors:** Hiroki Takeuchi, Michiyo Iba, Haruhisa Inoue, Makoto Higuchi, Keizo Takao, Kayoko Tsukita, Yoshiko Karatsu, Yumiko Iwamoto, Tsuyoshi Miyakawa, Tetsuya Suhara, John Q. Trojanowski, Virginia M. -Y. Lee, Ryosuke Takahashi

**Affiliations:** 1 Department of Neurology, Graduate School of Medicine, Kyoto University, Sakyo-ku, Kyoto, Japan; 2 Center for iPS Cell Research and Application (CiRA), Kyoto University, Sakyo-ku, Kyoto, Japan; 3 Center for Neurodegenerative Disease Research, Institute on Aging and Department of Pathology and Laboratory Medicine, University of Pennsylvania School of Medicine, Hospital of the University of Pennsylvania (HUP), Philadelphia, Pennsylvania, United States of America; 4 Core Research for Evolutional Science and Technology (CREST), Japan Science and Technology Agency, Kawaguchi, Saitama, Japan; 5 Molecular Imaging Center, National Institute of Radiological Sciences, Chiba, Chiba, Japan; 6 Section for Behavior Patterns, Center for Genetic Analysis of Behavior, National Institute for Physiological Sciences, Okazaki, Aichi, Japan; 7 Division of Systems Medical Science, Institute for Comprehensive Medical Science, Fujita Health University, Toyoake, Aichi, Japan; Mental Health Research Institute of Victoria, Australia

## Abstract

Tauopathies are neurodegenerative disorders characterized by the accumulation of abnormal tau protein leading to cognitive and/or motor dysfunction. To understand the relationship between tau pathology and behavioral impairments, we comprehensively assessed behavioral abnormalities in a mouse tauopathy model expressing the human P301S mutant tau protein in the early stage of disease to detect its initial neurological manifestations. Behavioral abnormalities, shown by open field test, elevated plus-maze test, hot plate test, Y-maze test, Barnes maze test, Morris water maze test, and/or contextual fear conditioning test, recapitulated the neurological deficits of human tauopathies with dementia. Furthermore, we discovered that prepulse inhibition (PPI), a marker of sensorimotor gating, was enhanced in these animals concomitantly with initial neuropathological changes in associated brain regions. This finding provides evidence that our tauopathy mouse model displays neurofunctional abnormalities in prodromal stages of disease, since enhancement of PPI is characteristic of amnestic mild cognitive impairment, a transitional stage between normal aging and dementia such as Alzheimer's disease (AD), in contrast with attenuated PPI in AD patients. Therefore, assessment of sensorimotor gating could be used to detect the earliest manifestations of tauopathies exemplified by prodromal AD, in which abnormal tau protein may play critical roles in the onset of neuronal dysfunctions.

## Introduction

The microtubule-associated protein tau is mainly expressed in the central nervous system. Tau protein binds to tubulin in microtubules, which is a major component of the cytoskeleton and where it promotes their polymerization and stabilization [1], [2]. Mutated and/or hyperphosphorylated tau accumulates in the disease state, where it is thought to contribute to neuronal cell death [3], [4]. Neurodegenerative disorders with abnormal tau protein depositions are called tauopathies, because they are characterized by tau inclusions such neurofibrillary tangle (NFT) in Alzheimer's disease (AD), and Pick bodies in Pick's disease [5]–[9]. In the brains of patients with hereditary tauopathy, frontotemporal dementia and parkinsonism linked to chromosome 17 (FTDP-17), mutant tau proteins are aberrantly hyperphosphorylated and less soluble than wild type tau [10]–[13]. Several tauopathy mouse models have contributed to the novel pathophysiological findings of tauopathies, including behavioral abnormalities [14]. However, early behavioral symptoms of tauopathy model mice have not yet been evaluated in detail. We previously analyzed the brains of a mouse model of tauopathy expressing mutant human tau gene (P301S, 1N4R), one of the mutations in human FTDP-17 [15]. These transgenic mice closely model human tau pathology seen in authentic tauopathies. For example, they develop synaptic pathology at 3 months of age, filamentous tau lesions at 6 months of age, and progressive tau accumulations similar to NFTs in association with neuronal loss as well as hippocampal and entorhinal cortical atrophy by 9–12 months of age [15]. Here, we evaluated the behavioral phenotypes of the tauopathy mouse model from 3 months of age, before they developed NFT-like tau pathology, neuronal cell death and motor weakness, and delineated a novel behavioral phenotype, increased prepulse inhibition (PPI), that is observed in the prodromal stage of AD, at the earliest stages of disease onset in our tauopathy model mice.

## Results

Table 1 shows a summary of the tests/tasks used in this study, anatomical structures related to the tests/tasks, and related behavioral/cognitive functions.

**Table 1:** Performed individual tasks, their estimated affected brain regions, and related behavioral abnormalities.

Tasks	Affected regions	Related behavioral abnormalities
Righting reflex	Vestibular system, muscle	Postural maintenance, Muscle strength
Whisker twitch	Sensory system	Tactile sensation
Ear twitch	Sensory system	Tactile sensation
Reaching	Visual system	Visual acuity
Key jangling	Auditory system	Hearing acuity
Grip strength test	Motor system	Muscle strength
Wire hang test	Motor system	Muscle strength
Light/Dark transition test	Cingulate cortex	Anxiety
Open field test	Cingulate cortex	Anxiety, Exploratory locomotion
Elevated plus maze	Cingulate cortex	Anxiety
Porsolt forced swim test	Stria terminalis (basal forebrain)	Behavioral despair
Social interaction test	Hippocampus	Anxiety in novel situation, Sociality
Rotarod treadmill test	Hippocampus, Motor tract, Cerebellum	Learning, Muscle weakness, Motor activity, Coordination
Hot plate test	Supraspinal sensory tract	Pain and temperature sensation
Prepulse inhibition test	Prefrontal cortexHippocampusStriatum	Sensorimotor gating, Startle response, Hearing acuity
Y-maze	Hippocampus	Spontaneous alternation behavior, Learning (cognitive function)
Crawley's social interaction test	Hippocampus	Reference memory (cognitive function), Sociality
Barnes circular maze	Hippocampus	Spatial working memory, Reference memory (cognitive function)
Morris water maze	Hippocampus	Spatial working memory, Reference memory (cognitive function)
Contextual and cued fear conditioning	Hippocampus and Amygdala	Contextual memory (cognitive function)
Tail suspension test	Stria terminalis (basal forebrain)	Behavioral despair

Table legend. Performed individual tasks. Tauopathy model mice had normal fur, whiskers and posture, and were indistinguishable from wild type mice. Casual trials of righting reflex, whisker touch reflex, ear twitch reflex, reaching, and key jangling test were also normal.

### No significant differences in general conditions and neurological screening

Upon gross inspection, tauopathy model mice had normal fur, whiskers and posture, and were indistinguishable from wild type mice. Casual trials of righting reflex, whisker touch reflex, ear twitch reflex, reaching, and key jangling test were also normal. They had similar body weight and body temperature (Figure S2A and S2B). In grip-strength and wire-hang tests, their performances were also similar, indicating that general muscular functions of tauopathy model mice were not significantly impaired at 3 months of age (Figure S2C and S2D).

### No significant differences in light/dark transition test

In the light/dark transition test, no statistically significant differences were observed between tauopathy model mice and wild type mice (Figure S3A–C). However, latency to enter the light chamber tended to be decreased in the former (Figure S3D).

### Increased hyperactivity and decreased anxiety-like behavior in tauopathy model mice

**Figure 1:**
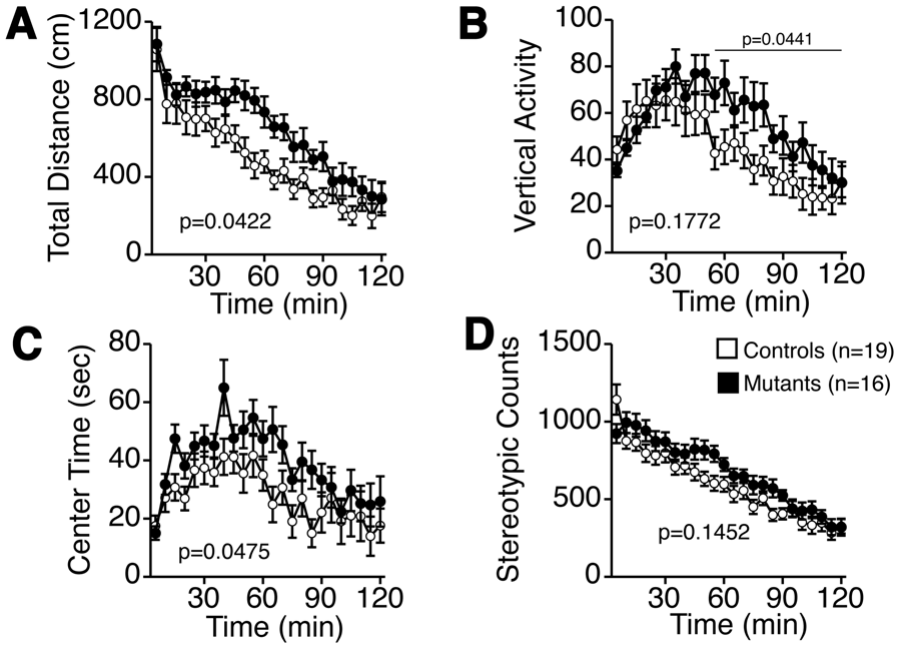
Open field test in 13-week-old mice. Total distance traveled (A), vertical activity (B), center time (C), stereotypic counts (D). Tauopathy model mice traveled longer distance (*F*(1, 33) = 4.467, *p* = 0.0422, between groups, interaction between genotypes and time; *F*(1,23) = 1.955, *p* = 0.0049), showed more vertical activity in the latter half of the task (*F*(1, 33) = 4.382, *p* = 0.0441, between groups), spent more time in the center of the field (*F*(1, 33) = 4.239, *p* = 0.0475, between groups), significantly. No statistical significance was observed in stereotypic counts (*F*(1, 33) = 2.226, *p* = 0.1452, between groups). Controls: wild type mice (n = 19); Mutants: tauopathy model mice (n = 16). Tested with two-way mixed model ANOVA.

**Figure 2:**
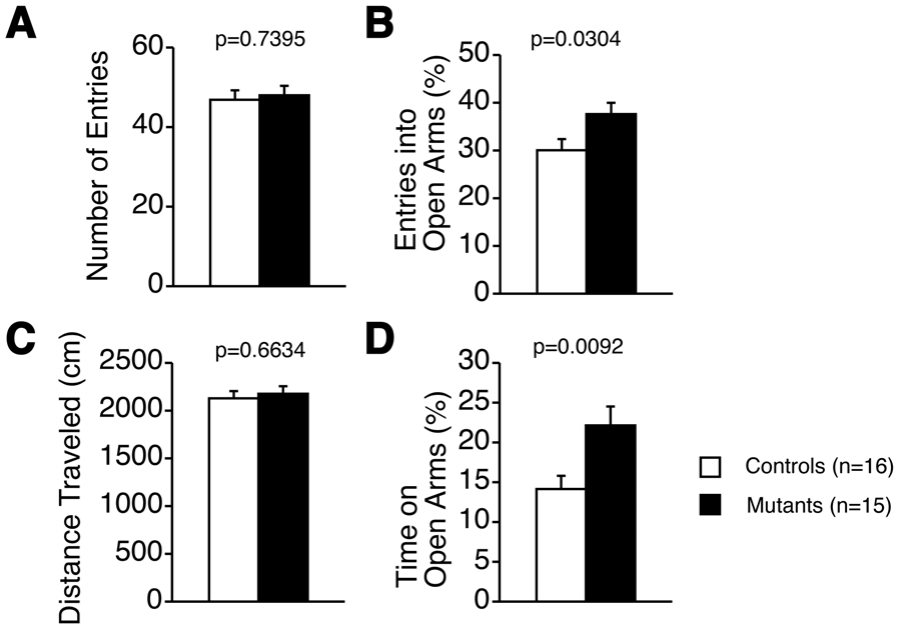
Elevated plus maze test. Number of total entries (A), percentage of entries into open arms (B), total distance traveled during the test (C), percentage of time on open arms (D). Percentages of entries into open arms and of time in open arms were significantly increased in tauopathy model mice, respectively. Three wild type mice and 2 tauopathy model mice dropped from the open arms and failed to complete the task. Controls: wild type mice (n = 16); Mutants: tauopathy model mice (n = 15). Tested with Student's *t*-test.

We then applied the open field test paradigm to explore anxiety-like behavior (Figure 1A–D). Tauopathy model mice showed an increasing tendency in total locomotive distance (Figure 1A), and vertical activity was increased in the latter half of the test (Figure 1B). Time spent in the center of the field was also increased (Figure 1C). Stereotypic locomotion tended to be increased (Figure 1D). In the elevated plus-maze test, total entries and total distance traveled were not significantly different between the two groups of mice (Figure 2A, C). However, both entries into and time spent on the open arms were significantly increased in tauopathy model mice (Figure 2B, D). Therefore, anxiety-like behavior was reduced in tauopathy model mice compared to wild type mice, and tauopathy model mice were more hyperactive.

### Locomotor activity and motor coordination

In the rotarod test, tauopathy model mice demonstrated no significant change in latencies to fall compared with wild type mice (Figure S4A). Since the effect of motor learning reached a plateau in wild type mice after the 4^th^ trial, we also analyzed the performance of each genotype in the 4^th^-6^th^ trials. In these trials, there was also no significant difference in latency to fall. Therefore, no difference was observed between locomotor activity and motor coordination.

### Increased antinociceptive responses of tauopathy model mice

We next tested antinociceptive responses by conducting the hot plate test. Tauopathy model mice exhibited significantly decreased thresholds in the hot plate test (Figure S4B). As the hot plate response is considered to involve supraspinal lesions [16], the supraspinal sensory tract might be affected in brains of tauopathy model mice.

### Behavioral despair of tauopathy model mice

**Figure 3:**
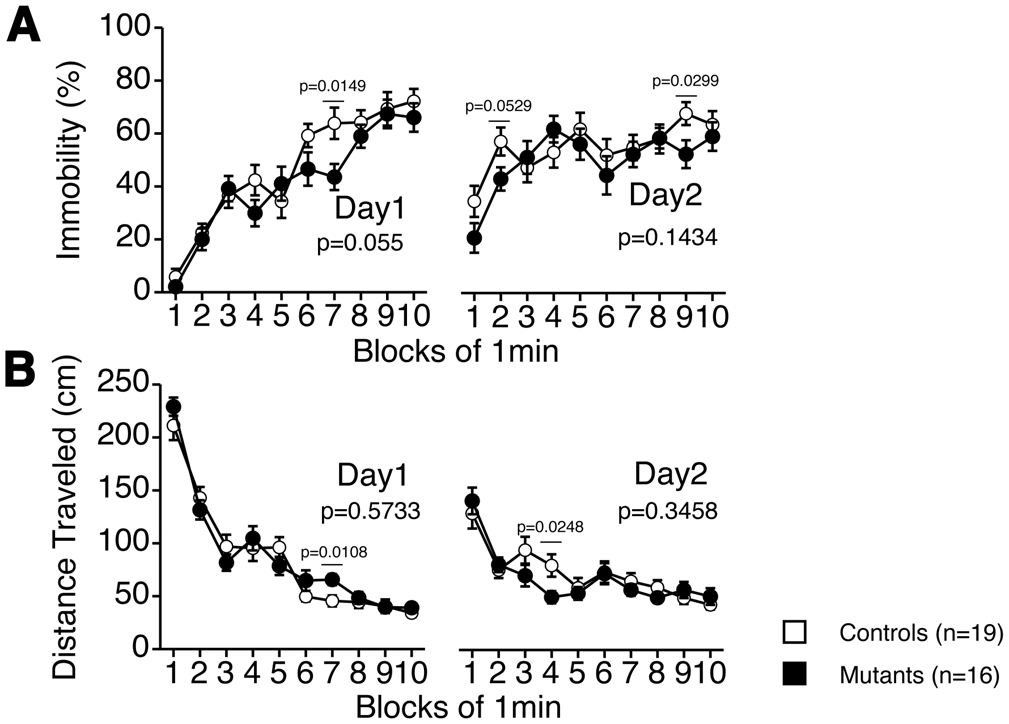
Porsolt forced swim test. Percentage of each minute immobilized (A), distance traveled during the test (B). In the 6^th^ block of Day 1, immobility was decreased (two-way mixed model ANOVA, *F*(1, 33) = 6.607, *p* = 0.0149) and distance traveled was increased in tauopathy model mice (two-way mixed model ANOVA, *F*(1, 33) = 7.308, *p* = 0.0108). In general, no statistical significance was observed, tested with two-way mixed model ANOVA (immobility: Day 1, *F*(1, 33) = 3.958, *p* = 0.055, between groups. Day 2, *F*(1, 33) = 2.246, *p* = 0.1434, between groups; distance traveled: Day 1, *F*(1, 33) = 0.324, *p* = 0.5733, between groups. Day 2, *F*(1, 33) = 0.915, *p* = 0.3458, between groups). Controls: wild type mice (n = 19); Mutants: tauopathy model mice (n = 16).

In a portion of the observed period (7^th^ min, Day 1) of the Porsolt forced swim test, tauopathy model mice showed decreased immobility time and increased distance traveled (Figure 3A, B). However, generally, no statistical significance was observed in immobility between tauopathy model mice and wild type mice in this test (*F*(1, 33) = 3.958, *p* = 0.055 on Day 1, *F*(1, 33) = 2.246, *p* = 0.1434 on Day 2) and in the tail suspension test (*F*(1, 32) = 0.884, *p* = 0.3542, Figure S5).

### Sensorimotor gating

**Figure 4:**
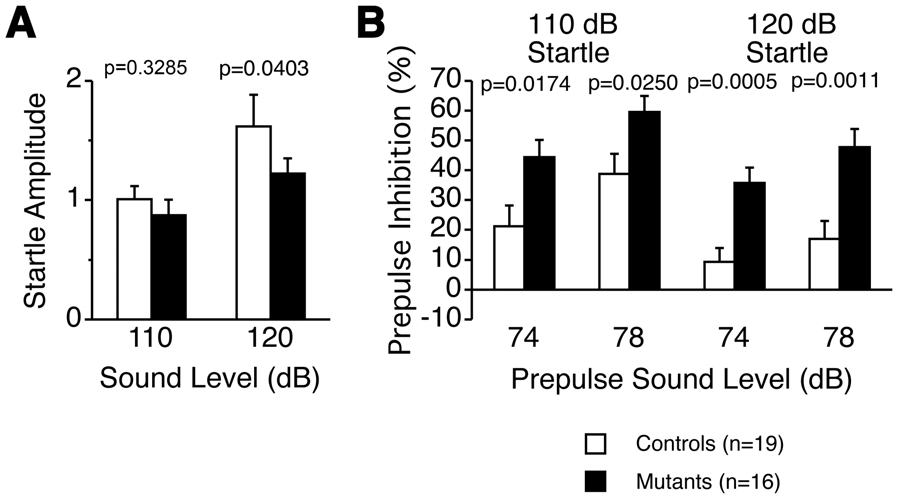
Prepulse inhibition test. Startle amplitude (A), percentage of prepulse inhibition (B). Tauopathy model mice had lower startle amplitudes than wild type mice at both 110 dB and 120 dB, statistically significant at 120 dB. Percent PPI was significantly greater in tauopathy model mice. Controls: wild type mice (n = 19); Mutants: tauopathy model mice (n = 16). Tested with Mann-Whitney's U-test in startle amplitude 110 dB, the others with Student's t-test.

The prepulse inhibition (PPI) test is widely used to measure deficits in information-processing abilities or sensorimotor gating in schizophrenia patients [17], and it can be employed in both human and animal experiments [18]. PPI is defined as the degree (%) by which the motor acoustic startle response is reduced when the startle-eliciting stimulus is preceded by a brief, low-intensity, non-eliciting stimulus. Tauopathy model mice had lower startle amplitudes than wild type mice at both 110 dB and 120 dB, and this was statistically significant at 120 dB (Figure 4A). Especially, the %PPI, an index of sensorimotor gating, was significantly greater in tauopathy model mice (Figure 4B). The reduced PPI values at 120 dB rather than those at 110 dB might be a ceiling effect caused by the strong intensity of the startle stimulus.

### Impaired spontaneous alternation in tauopathy model mice

**Figure 5:**
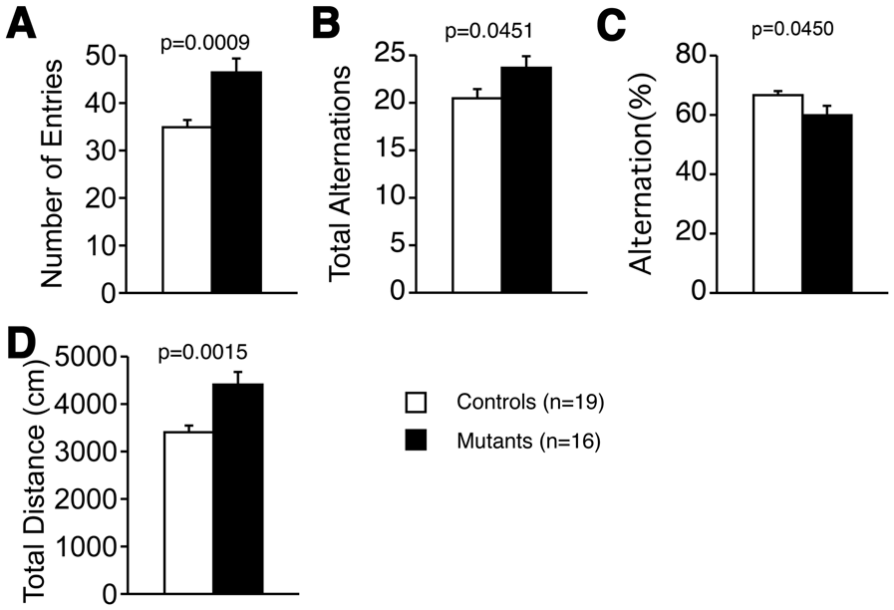
Y-maze test. Number of total entries (A), number of total alternations (B), total distance traveled (C), percentage of alternations (D). In tauopathy model mice, significantly increased numbers of total entries and total alternations, and prolonged distance traveled were observed. Percentage of alternations was significantly decreased, suggesting that short-term memory was impaired in tauopathy model mice. Controls: wild type mice (n = 19); Mutants: tauopathy model mice (n = 16). Tested with Mann-Whitney's U-test in % alternation, the others with Student's *t*-test.

We performed the Y-maze test to evaluate spontaneous alternative behavior. Tauopathy model mice demonstrated increased numbers of entries into each arm, total alternations, and total distance traveled (Figure 5A–C), reflecting hyperactivity. However, the percentage of alternations was significantly decreased (Figure 5D).

### Decreased sociability/object recognition memory in tauopathy model mice

During the social interaction test in a one-chamber novel environment, no statistical significance was observed in the time spent and the number of contacts, duration of active contacts, mean of duration/contact ratio, and distance traveled between the two groups of mice (Figure S6A–E).

**Figure 6:**
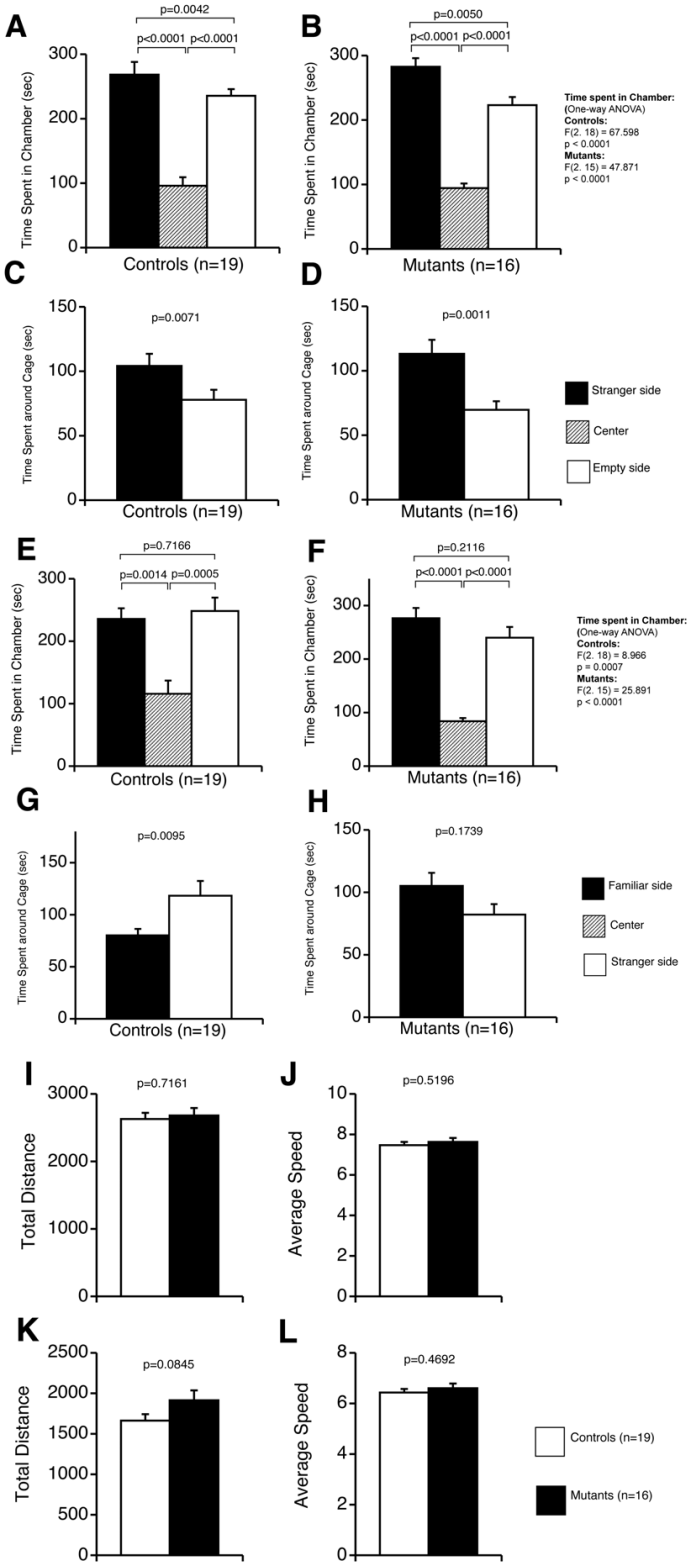
Crawley's social interaction test. The 1^st^ trial with one-stranger mouse (A–D, I, J), the 2^nd^ trial with one-familiar mouse and one-stranger mouse (E–H, K, L). Time spent in each chamber was tested with one-way ANOVA, followed by Fisher's least significant difference method as *post hoc* analysis within groups, with Student's t-test between groups. Time spent in each cage (mouse with no prior contact with a subject mouse) was analyzed by Student's *t*-test and Welch's t-test. In the 1^st^ trial, both groups spent more time in the chamber (A, B) and the cage (C, D) of the stranger side. In the 2^nd^ trial, no statistical significance was observed between the time spent in the stranger and familiar chambers of the two groups (Controls: *p* = 0.7166; Mutants: *p* = 0.2116, Controls vs Mutants: Stranger side: *p* = 0.5308, Empty side: *p* = 0.4412). Control wild type mice showed significantly more time spent with stranger mice (G, Controls vs Mutants: *p* = 0.03761 (Welch's t-test), Familiar side: *p* = 0.04053 (Student's t-test)). However, tauopathy model mice tended to spend more time with familiar mice (H). Controls: wild type mice (n = 19); Mutants: tauopathy model mice (n = 16).

Crawley's three-chamber social approach test consists of a sociability test and a social novelty preference test. These tests assess social interaction that is relatively independent of locomotor activity compared to other social interaction tests, because the preference of the mice can be quantified based on the time spent around a wire cage containing a stranger mouse vs. an empty cage in the sociability test, and a stranger mouse vs. a familiar mouse in the social novelty preference test [19]. No remarkable significance was observed during the habituation with the stranger mouse (Figure 6A–D), or in time spent in the stranger or familiar chamber (Figure 6E, F). Wild type mice spent significantly longer time with stranger mice (Figure 6G), whereas tauopathy model mice spent a shorter time with stranger mice than with familiar mice (Figure 6H). No statistical significance was observed in total distance traveled (Figure 6I, K) and in average speed (Figure 6J, L). We also assessed statistical significances between groups (Controls vs Mutants). No significance was observed in the 1^st^ trial and the time spent in each chamber in the 2^nd^ trial. Time spent around each cage showed significances between control mice and tauopathy model mice. Therefore, tauopathy model mice had some impaired sociability/object recognition memory.

### Spatial learning abilities

Spatial memory, which depends on the function of the hippocampus, was assessed by the Barnes circular maze test. The task is similar to the Morris water maze test, as both require an escape response.

All mice learned to locate the escape hole during the training period, as indicated by a progressive reduction in distances, latencies and numbers of escape errors. Through the training trials, there were no statistical differences between tauopathy model mice and wild type mice in distances, latencies, and errors (Figure S7A–C).

The probe trial was conducted 24 h after the last training session. Both tauopathy model mice and wild type mice selectively located the correct target hole where the escape box had been. However, tauopathy model mice tended to spend less time around the target hole (Figure S7D).

**Figure 7:**
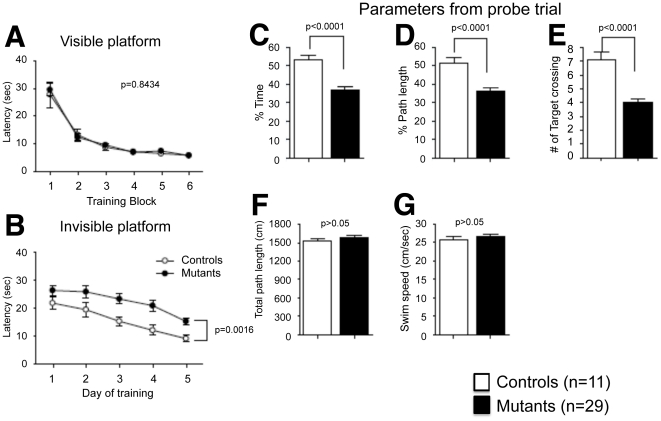
Morris water maze test. Training session (A, B), probe test 24 h after the last (6^th^) training (C–G). No statistical significances were observed in the training session with the visible platform (A, *F*(1,46) = 0.0395, *p* = 0.8434, between groups), but tauopathy model mice spent significantly more time to find the invisible platform (B, *F*(1,46) = 11.2326, *p* = 0.0016, between groups). Tested by two-way mixed model ANOVA. In the probe trial, tauopathy model mice showed less time (C, *p*<0.0001) and path length (D, *p*<0.0001) in the pool quadrant where the platform had previously been placed, and smaller numbers of target platform crossings (E, *p*<0.0001). No statistical significance was observed in total path length (F, *p*>0.05) and swimming speed (G, *p*>0.05) between the two groups of mice. Controls: wild type mice (n = 11); Mutants: tauopathy model mice (n = 29). Tested with Student's *t*-test.

Spatial memory was also assessed by the Morris water maze test to find reproducibility with another task. All mice learned to locate the platform during the training period, as indicated by a progressive reduction in distances, latencies and numbers of errors to escape. Through the training trials, there were no statistical differences between tauopathy model mice and wild type mice in latencies to find the visible platform (Figure 7A). However, tauopathy model mice spent significantly more time to find the invisible platform (Figure 7B), suggesting impairment of their spatial learning ability. During the probe trial, tauopathy model mice showed less time and path length in the pool quadrant where the platform had previously been placed (Figure 7C, D), and a smaller number of target platform crossings (Figure 7E). These results of the probe trial suggest that spatial memory was impaired in tauopathy model mice. No statistical significance was observed in total path length and swimming speed of the two groups of mice, implying that swimming ability did not bias the results of training tasks or the probe trial (Figure 7F, G).

### Contextual memory

**Figure 8:**
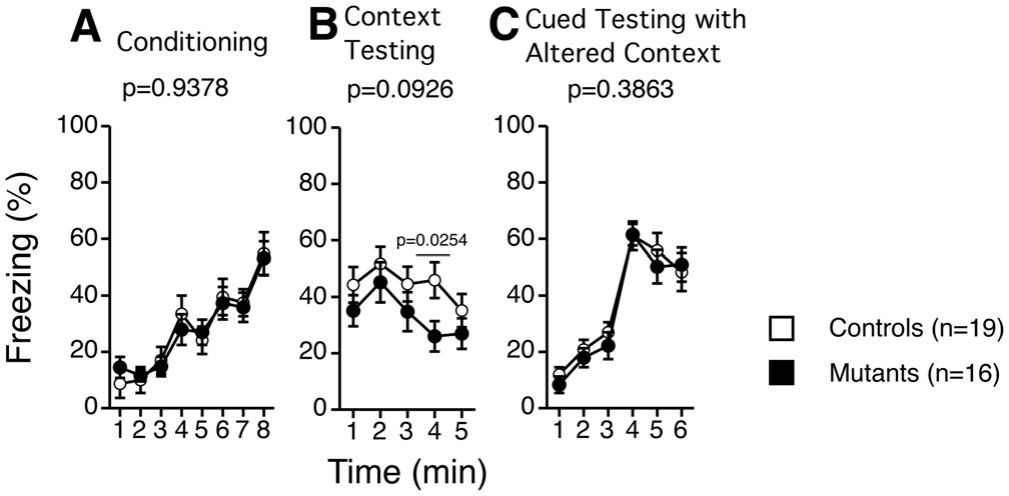
Fear conditioning test. Percentage of freezing time of each minute of conditioning (A), context testing (B), cued testing with altered context (C). Freezing tended to be reduced in tauopathy model mice in context testing. In the 4^th^ block, freezing was significantly decreased (two-way mixed model ANOVA, *F*(1, 33) = 5.479, *p* = 0.0254). In general, no statistical significance was observed as tested with two-way mixed model ANOVA (Conditioning: *F*(1, 33) = 0.006, *p* = 0.9378, between groups; Context testing: *F*(1, 33) = 3.000, *p* = 0.0926, between groups; Cued testing: *F*(1, 33) = 0.771, *p* = 0.3863, between groups). Controls: wild type mice (n = 19); Mutants: tauopathy model mice (n = 16).

We also performed contextual and cued fear conditioning tests, which are largely dependent on the hippocampus and amygdala [20], [21]. These tests did not show any differences in response between the two groups (Figure 8A–C) except for the fourth time point in the context testing (*p* = 0.0254, Figure 8B), which showed decreased immobility in tauopathy model mice. These results indicate that the effect of mutant tau protein on learning in the contextual fear conditioning test was minimal around the age of 5–6 months.

### Hyperphosphorylated Tau expression in the brains of tauopathy model mice

**Figure 9:**
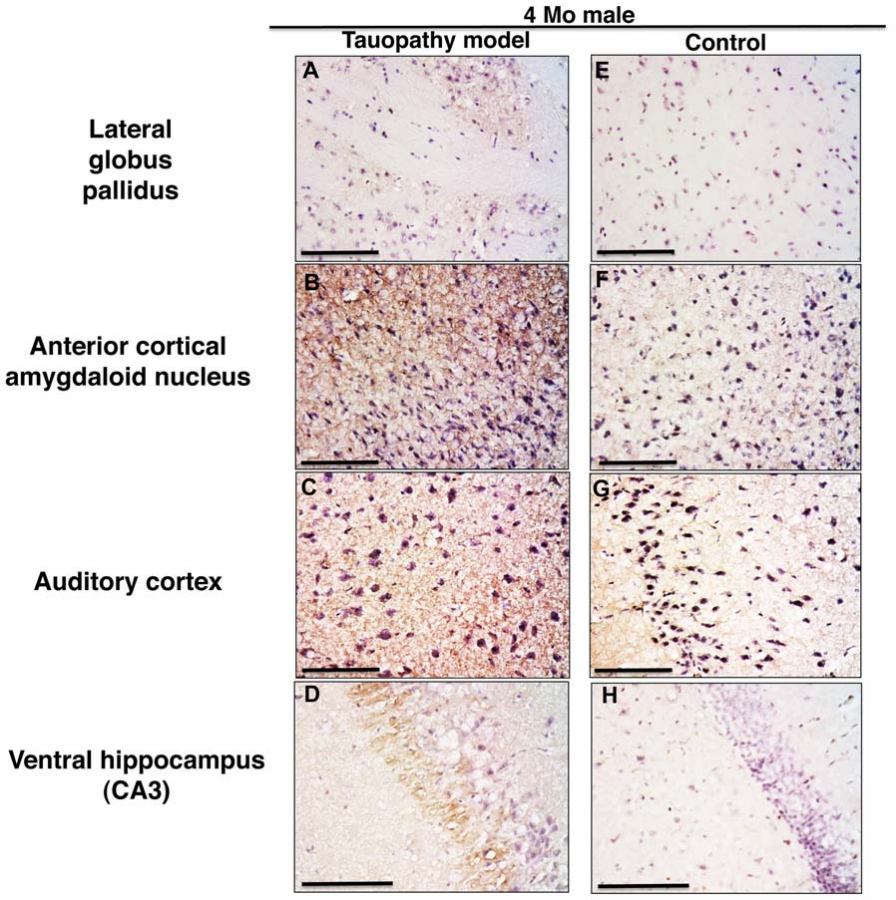
Representative immunofluorescence histological findings of tauopathy model mice and wild type mice. Male mice 4 months of age: Tauopathy model mice (A–D), and wild type mice (E–H). Lateral globus pallidus (A, E), anterior cortical amygdaloid nucleus (B, F), auditory cortex (C, G), and ventral hippocampus (CA3; D, H). Scale Bar: 100 µm.

**Figure 10:**
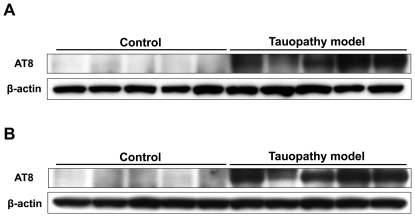
Representative immunoblotting of the whole cortex (A) and the whole hippocampus (B) of mice at the age of 6 months, after the comprehensive behavioral analysis. Phosphorylated tau was observed in the brains of tauopathy model mice, both in the cortex and in the hippocampus, but not in those of control mice. Each 5 samples were randomly selected.

At 4 months of age, we assessed the histological findings of the lateral globus pallidus, amygdala, auditory cortex, and ventral hippocampus of male tauopathy model mice. These regions are thought to be affected by sensorimotor gating. Phosphorylated tau was observed in these regions of tauopathy model mice (Figure 9A–D), but not in wild type mice (Figure 9E–H). After behavioral analysis, we also assessed the histological findings of the cingulate cortex, anterior cortical amygdaloid nucleus and dorsal hippocampus. The cingulate cortex and amygdala are considered to be affected by anxiety disorder [22]. Dorsal hippocampal lesions are closely related to memory disturbance [23]. Remarkable phosphorylated tau was observed in these three regions of tauopathy model mice, but not in wild type mice, corresponding to the previous report [15], suggesting that tau pathology is related to the observed behavioral abnormalities (data not shown). Immunoblotting analysis revealed the expression of phosphorylated tau in the hippocampus and cortex of tauopathy model mice at 6 months of age (Figure 10), also compatible with the previous reports [15], [24], [25].

## Discussion

We previously reported that our tauopathy model mice closely recapitulated human tau pathology including presynaptic loss, filamentous tau lesions, and progressive tau accumulations similar to NFTs seen in human tauopathy [15]. Behavioral analysis of this mouse model, therefore, would demonstrate prodromal tauopathy.

We demonstrated that PPI was enhanced in early-stage tauopathy model mice. Previous studies showed abnormal phenotypes in multiple behavioral batteries [23], but PPI enhancement in the early stage had not been observed in previous studies.

Synaptic loss was one of the pathological findings in the brains of these tauopathy model mice [15]. A previous study reported decreased PPI in human N279K tauopathy model mice older than 12 months, with neuronal cell death [26]–[28]. They speculated that decreased PPI was related to neuronal cell death. In contrast, PPI enhancement in our tauopathy model mice would be due to presynaptic loss [15] caused by profound neurotoxicities prior to the emergence of high-order tau assemblies [29], or by aggregation of abnormal protein itself [30].

PPI is related to the circuit composed of the colliculus, pedunculopontine tegmental nucleus, substantia nigra, and caudal pontine reticular nucleus. This circuit is modulated by some structures of the forebrain — auditory cortex, amygdala, lateral globus pallidus, and ventral hippocampus [31]–[40]. The abnormal tau protein accumulation we observed in the early stage of tauopathy (Figure 9, 10) might stimulate neurons in the amygdala and ventral hippocampus, leading to the enhancement of PPI.

Patients with mild cognitive impairment (MCI), the boundary or transitional stage between normal aging and dementia including AD, revealed greater %PPI than age-matched normal subjects, although patients with moderate-stage and advanced AD showed lower %PPI [41], [42]. Therefore, it would seem reasonable for PPI to show no difference between AD patients and normal subjects during the progression of cognitive dysfunction [43]. We observed enhancement of PPI, and decreased PPI with old (older than 12 months) tauopathy model mice has been previously reported [26]–[28]. PPI with our tauopathy model mice at an older age might also be decreased, although we were prevented from performing PPI assay with our tauopathy model mice, being older than 6 months, because of motor weakness. Therefore, the behavioral abnormalities we observed with our mice might be helpful for assessing prodromal human AD, and especially the relationship between its tau pathology and cognitive dysfunction. These findings suggest that enhancement of PPI could be one of the early manifestations in tauopathies including AD, and tau protein abnormalities may be relevant to MCI.

Locomotor hyperactivity was observed in the open field test, as previously reported [26], [44], Y-maze test, and Morris water maze test. Motor weakness was observed after 6 months of age [15], but we failed to find lower grip strength (Figure S1C). Therefore, the motor tract of these tauopathy model mice was functionally intact at 3 months of age. Our tauopathy model mice showed phosphorylated tau in the prefrontal cortex and amygdala [15], which are involved in hyperactive behavior [45]. Hyperactive behavior was also observed among human AD patients [46]. Thus, our tauopathy model mice appeared to reproduce the hyperactive phenotype of tauopathy with dementia. Our mice also showed less latency in the hot plate test, an antinociceptive reaction assessment, suggesting that these mice were more sensitive to pain and their supraspinal sensory tracts were impaired. However, patients with dementia showed more impaired response against pain [47]. Hyperactivity might modify the results of the hot plate test.

Despair or depressive states are often observed in patients with dementia [48]. However, such patients also frequently present with agitation and aggression [49]. Our study showed partially reduced immobility in tauopathy model mice in the Porsolt forced swim test, suggesting that despair-like behavior might be decreased and some part of agitation in patients with cognitive dysfunction reproduced. Prolonged time spent in the center of the open field or open arms in the elevated plus test was observed, suggesting a reduction in anxiety-like behavior. The cingulate cortex and/or amygdala might be involved in impaired anxiety, which would correspond to patients with AD [50] and/or frontotemporal dementia [51].

Our battery, including the Y-maze test, Crawley's social interaction test, Barnes maze test, Morris water maze test, and context testing in the fear conditioning test disclosed memory disturbance in our tauopathy model mice. This is compatible with human cognitive disorders, including AD, that dorsal hippocampal lesion affects memory disturbance [52]–[54]. The Barnes maze test was chosen for this study, as it does not involve swimming like the Morris water maze. Given the possible motor deficits with tauopathy model mice, swimming ability might have given an advantage to wild type mice in the Morris water maze. However, only the Morris water maze test showed statistical significance in tasks concerning spatial memory, suggesting that this test might in fact be more effective than the Barnes maze test to motivate mice to find the target as they try to avoid drowning. Female mice might show more remarkable behavioral abnormalities. Further studies will be required to confirm the effect on memory disturbance by gender and/or background.

In conclusion, human P301S tau protein transgenic mice could successfully recapitulate symptoms of human tauopathies with dementia, including AD at the early stage. Behavioral analysis in the earlier stage of tauopathy model mice might provide new avenues toward early diagnosis and early therapeutic intervention for tauopathies including AD.

## Materials and Methods

### Animals

We have previously established transgenic mice expressing the mutant (P301S) human T34 tau isoform (1N4R) on a B6C3H/F1 background [15]. Six-month-old animals with this original hybrid background were used for the Morris water maze test. All the other animals were backcrossed 10 times to C57BL/6J background to create a congenic strain. Transgenic and non-transgenic offspring were identified by PCR of tail DNA.

### Experimental design

Six-month-old female animals with a B6C3H/F1 background (tauopathy model mice: n = 29; littermate wild type mice: n = 11) were used to perform the Morris water maze test. All other studied animals were male with a C57BL/6J background, and in each individual test, tauopathy model mice (n = 16) were compared with littermate wild type mice (n = 19). Behavioral tests were evaluated in the same animals. Three or four mice were housed in each cage in a room with a 12-hour light/dark cycle (lights on at 7:00 a.m.) with access to food and water *ad libitum*. Behavioral testing was performed between 9:00 a.m. and 6:00 p.m. After the tests, the apparatuses were cleaned with super hypochlorous water to prevent any bias due to olfactory cues. All behavioral testing procedures were carried out as previously described [55], in accordance with the National Institutes of Health (NIH) *Guide for the Care and Use of Laboratory Animals*, and were approved by the Animal Care and Use Committee of Kyoto University Graduate School of Medicine (Permit number: Med Kyo 09567) and the Institutional Animal Care and Use Committee of University of Pennsylvania School of Medicine (Permit number: 507800). Each task was performed in series as described in Figure S1.

### General health and neurological screening

Neurological screening was performed with 13-week-old male mice. The righting, whisker twitch, and ear twitch reflexes were evaluated. A number of physical features, including the presence of whiskers and bald hair patches, were also recorded.

### Neuromuscular strength

Examinations of neuromuscular strength were performed with 13-week-old male mice, and tested with the grip strength test and wire hang test. A grip strength meter (O'Hara & Co., Tokyo, Japan) was used to assess forelimb grip strength. Mice were lifted and held by their tail so that their forepaws could grasp a wire grid. The mice were then gently pulled backward by the tail with their posture parallel to the surface of the table until they released the grid. The peak force applied by the forelimbs of the mouse was recorded in Newtons (N). Each mouse was tested three times, and the greatest value measured was used for statistical analysis. In the wire hang test, the mouse was placed on a wire mesh that was then inverted and waved gently, so that the mouse gripped the wire. Latency to fall (in sec) was recorded, with a 60-sec cut-off time.

### Open field test

Locomotor activity was measured using the open field test, which was performed with 14-week-old male mice. Each mouse was placed in the center of the open field apparatus (40×40×30 cm; Accuscan Instruments, Columbus, OH, USA). Total distance traveled (in cm), vertical activity (rearing measured by counting the number of photobeam interruptions), time spent in the center, beam-break counts for stereotyped behaviors, and number of fecal boli were recorded. Data were collected for 120 min.

### Light/dark transition test

Light/dark transition test was performed as previously described [56]. The apparatus used consisted of a cage (21×42×25 cm) divided into two chambers of equal size by a partition containing a door (O'Hara & Co.). One chamber was brightly illuminated (390 lux), whereas the other was dark (2 lux). Mice were placed into the dark side and allowed to move freely between the two chambers with the door open for 10 min. The total number of transitions between chambers, time spent in each side, first latency to enter the light side, and distance traveled were recorded automatically using Image LD software (see ‘Image and data analysis’).

### Elevated plus-maze test

Elevated plus-maze test was performed as previously described [57]. The elevated plus-maze (O'Hara & Co.) consisted of two open arms (25×5 cm) and two enclosed arms of the same size, with 15-cm high transparent walls. The arms and central square were made of white plastic plates and were elevated to a height of 55 cm above the floor. To minimize the likelihood of animals falling from the apparatus, 3-mm high plastic ledges were provided for the open arms. Arms of the same type were arranged at opposite sides to each other. Each mouse was placed in the central square of the maze (5×5 cm), facing one of the closed arms. Mouse behavior was recorded during a 10-min test period. The numbers of entries into, and the time spent in open and enclosed arms, were recorded. The illumination level was 100 lux at the center of the maze. For data analysis, we used the following four measures: the percentage of entries into the open arms, time spent in the open arms (sec), number of total entries, and total distance traveled (cm). Data acquisition and analysis were performed automatically using Image EP software (see ‘Image and data analysis’). Three wild type mice and a tauopathy model mouse dropped from the open arms and failed to finish the task.

### Porsolt forced swim test

The Porsolt forced swim test apparatus consisted of four Plexiglas cylinders (20 cm high×10 cm diameter). A nontransparent panel separated the cylinders to prevent the mice from seeing each other (O'Hara & Co.). The cylinders were filled with water (23°C) up to a height of 7.5 cm. Mice were placed into the cylinders, and their behavior was recorded over a 10-min test period (Day 1). Retention tests were administered 24 hours after training (Day 2). Data acquisition and analysis were performed automatically using Image PS software (see ‘Image and data analysis’).

### Hot plate test

The hot plate test for nociception was used to evaluate sensitivity to a thermal stimulus. Mice were placed on a 55.0 (±0.3) °C hot plate (Columbus Instruments, Columbus, OH, USA), and latency to the first hind-paw response (a foot shake or a paw lick) was recorded.

### Social interaction test in a novel environment (one-chamber social interaction test)

In the social interaction test, two mice of identical genotypes that were previously housed in different cages were placed in a box together (40×40×30 cm) and allowed to explore freely for 10 min. Social behavior was monitored with a CCD camera connected to a computer. Analysis was performed automatically using Image SI software (see ‘Image and data analysis’). The total number of contacts, total duration of active contacts, total contact duration, mean duration per contact, and total distance traveled were measured. Active contact was defined as follows. Images were captured at 1 frame per second, and distance traveled between two successive frames was calculated for each mouse. If the two mice contacted each other and the distance traveled by either mouse was longer than 2 cm, the behavior was considered as ‘active contact’. Genotypic mismatch was found in one pair (one tauopathy model pair), and they could not be analyzed.

### Rotarod test

Motor coordination and balance were tested by rotarod test. This test, using an accelerating rotarod (UGO Basile Accelerating Rotarod, Varese, Italy) with 5 lanes, was performed by placing 15-week-old mice on rotating drums (3-cm diameter) and measuring how long each animal was able to maintain its balance on the rod. The speed of the rotarod was accelerated from 4 to 40 rpm over a 5-min period. We performed 3 trials on the first day, and another 3 trials on the second day.

### Startle response/Prepulse inhibition (PPI) test

An acoustic startle reflex measurement system was used (O'Hara & Co.). A test session was begun by placing a 16-week-old mouse in a Plexiglas cylinder, where it was left undisturbed for 10 min. The duration of white noise, used as the startle stimulus, was 40 ms for all trial types. The startle response was recorded for 140 ms (measuring response every 1 ms) starting with the onset of the prepulse stimulus. The background noise level in each chamber was 70 dB. The peak startle amplitude recorded during the 140-ms sampling window was used as dependent variable. A test session consisted of 6 trial types (i.e., two types for startle stimulus-only trials, and four types for PPI trials). Intensity of the acoustic startle stimulus was 110 or 120 dB. The prepulse sound was presented 100 ms before the startle stimulus, and its intensity was 74 or 78 dB. Four combinations of prepulse and startle stimuli were employed (74–110 dB, 78–110 dB, 74–120 dB, and 78–120 dB). Six blocks of the 6 trial types were presented in a pseudorandom order such that each trial type was presented once within a block. The average inter-trial interval was 15 sec (range, 10–20 sec). Prepulse inhibition was defined as the percentage of the decline of startle response (prepulse inhibition (%) = 100−[(startle amplitude after prepulse and pulse)/(startle amplitude after pulse only)×100]).

### Y-maze test

We recorded spontaneous alternation behavior in a Y-maze to assess short-term memory performance [58]. The maze was made of gray painted wood. Each arm was 40 cm long, 13 cm high, 3 cm wide at the bottom, 10 cm wide at the top, and converged at an equal angle. The mouse was placed at the end of one arm and allowed to move freely through the maze during a 10-min session. The series of arm entries, including possible returns into the same arm, was recorded with a CCD camera connected to a computer. An alternation was defined as entries into all three arms on consecutive occasions. The number of maximum alternations was therefore the total number of arm entries minus two, and the percentage of alternations was calculated as (actual alternations/maximum alternations)×100. For example, if the arms were called A, B, C and the mouse performed ABCABCABBAB, the number of arm entries would be 11, and the successive alternations: ABC, BCA, CAB, ABC, BCA, CAB. Therefore, the percentage of alternations would be [6/(11−2)]×100 = 66.7. Analysis was performed automatically using Image YM software (see ‘Image and data analysis’).

### Crawley's sociability and preference for social novelty test

The test for sociability and preference for social novelty was conducted as previously described [19], [59], [60]. The apparatus comprised a rectangular, three-chambered box and a lid containing an infrared video camera (O'Hara & Co.). Each chamber measured 20×40×22 cm, and the dividing walls were made of clear Plexiglas, with small square openings (5×3 cm) allowing access into each chamber. An unfamiliar C57BL/6J male (stranger 1) with no prior contact with the subject mouse was placed in one of the side chambers. The placement of stranger 1 in the left or right side chamber was systematically alternated between trials. The stranger mouse was enclosed in a small, circular wire cage that allowed nose contact between the bars, but prevented fighting. The cage was 11 cm high, with a bottom diameter of 9 cm and bars spaced 0.5 cm apart. The subject mouse was first placed in the middle chamber and allowed to explore the entire social test box for 10 min. The amount of time spent within a 5-cm distance of the wire cage and in each chamber was recorded. At the end of the first 10 min, each mouse was tested in a second 10-min session to quantify social preference for a new stranger. A second, unfamiliar mouse was placed in the chamber that had been empty during the first 10-min session. This second stranger was enclosed in an identical small wire cage. The test mouse had a choice between the first, already-investigated unfamiliar mouse (stranger 1), and the novel unfamiliar mouse (stranger 2). As described above, the amount of time spent within a 5-cm distance of each wire cage and in each chamber during the second 10-min session was recorded. The stranger mice used in this experiment were 8- to 12-week-old C57BL/6J male mice, not littermates. Analysis was performed automatically using Image CSI software (see ‘Image and data analysis’).

### Barnes circular maze test

The Barnes task was conducted on “dry land,” a white circular surface, 1.0 m in diameter, with 12 holes equally spaced around the perimeter (O'Hara & Co.). The circular open field was elevated 75 cm from the floor. A black Plexiglas escape box (17×13×7 cm), which had paper cage bedding on its bottom, was located under one of the holes. The hole above the escape box represented the target, analogous to the hidden platform in the Morris task. The location of the target was consistent for any given mouse, but was randomized across mice. The maze was rotated daily, with the spatial location of the target unchanged with respect to the visual room cues, to prevent a bias based on olfactory or proximal cues within the maze. The first training was started when mice were 20 weeks old. Three trials per day were conducted for 10 successive days in the beginning (on days 5 and 6, no trial was undertaken). One day after the last training, a probe trial was conducted without the escape box, to confirm that this spatial task was acquired based on navigation using distal-environment room cues. Time of latency to reach the target hole, number of errors, distance to reach the target hole, and time spent around each hole were recorded by video tracking software (Image BM, see ‘Image and data analysis’).

### Morris water maze test

A circular pool (120 cm in diameter and 75 cm in height) was filled to a height of 30 cm with water (21±1°C), in which white tempera nontoxic paint was mixed to make the surface opaque. Mice were handled for two min, two times per day, for 2 days prior to pre-training. During the visible platform test, a black colored platform with a flag (10×10 cm in area) was placed in the quadrant 1 cm above the surface of the water, and its location was always varied randomly in each trial. All mice were subjected to 2 blocks divided by 4–5 hours per day with 3 trials per block for 3 consecutive days. For the invisible platform test, a white colored platform (10×10 cm in area) was placed at the center in one of four quadrants of the pool (southwest area) and submerged 1 cm below the water surface so that it was invisible at water level. The location of the platform was fixed at the same quadrant while the start position of swimming was varied. Mice were given 4 trials per day for 5 consecutive days, during which they were allowed to find the platform within 60 seconds. Each trial was separated by an inter-trial interval of 1–2 min, which was adopted through all the tests. Once the mouse located the platform, it was permitted to stay on it for 10 seconds. If the mouse did not find the platform within 60 seconds, it was guided to the platform and placed on it for 20 seconds. As parameters, escape latency (sec), swim path length (cm), and swim speed (cm/sec) were extracted from the recording data and averaged for each session of the trials and for each mouse. To evaluate the spatial reference memory 24 hours after the last trial of the invisible training test, all mice were given a probe trial consisting of removing the platform from the pool and allowing the mice to swim for 60 sec in its search. A record was kept of the swimming time (sec) in the pool quadrant where the platform had previously been placed. Swim speed (cm/sec), latency time to find the platform (sec), and the number of target-platform crossings during mouse swimming from the pool periphery to the pool quadrant were recorded by video camera and analyzed by a computer-controlled video tracking system (Smart V2.5, San Diego Inc., San Diego, CA, USA).

### Contextual and cued fear conditioning test

The test for contextual and cued fear conditioning was conducted as previously described [61]. Each mouse was placed in a test chamber (26×34×29 cm) inside a sound-attenuated chamber (O'Hara & Co.) and allowed to explore freely for 2 min. A 60-dB white noise, the conditioned stimulus (CS), was presented for 30 sec, followed by a mild (2 sec, 0.5 mA) foot shock, the unconditioned stimulus (US). Two more CS–US pairings were presented with 2-min interstimulus intervals. To examine shock sensitivity, we measured the distance traveled when the foot shock was delivered (from 2 sec before shock to 2 sec after, total 6 sec). Context testing was conducted 1 day after conditioning in the same chamber. Cued testing with altered context was conducted on the same day, following the context testing, using a triangular box (35×35×40 cm) made of white opaque Plexiglas, which was located in a different room. Data acquisition, control of stimuli (i.e., tones and shocks), and data analysis were performed automatically. Images were captured at one frame per sec. For each pair of successive frames, the size of the area (pixels) of mouse movement was measured. When this area was below a certain threshold (i.e., 20 pixels), the behavior was judged to be ‘freezing’. When the area equaled or exceeded the threshold, the behavior was considered ‘non-freezing’. The threshold (amount of pixels) for determining freezing was determined by adjusting it to the amount of freezing measured automatically, and ‘freezing’ that lasted <2 sec was not included in the analysis. Analysis was performed automatically using Image FZ software (see ‘Image and data analysis’).

### Tail suspension test

The tail suspension test was performed over a 10-min test session according to the procedures described previously [62]. Twenty-three-week-old mice were suspended 30 cm above the floor in a visually isolated area by adhesive tape placed ∼1 cm from the tip of the tail, and their behavior was recorded over a 10-min test period. Data acquisition and analysis were performed automatically using Image TS software (see ‘Image and data analysis’). One wild type mouse dropped and failed to finish the task.

### Immunohistochemistry

To evaluate the affected brain region in regard to sensorimotor gating, we assessed male tauopathy model mice at 4 months of age. Mice under deep pentobarbital anesthesia were perfused via the aorta with 50 mL of phosphate-buffered saline (PBS). After perfusion, the brain was quickly removed, followed by fixation with 4% paraformaldehyde in 100 mmol/L phosphate buffer (PB) for overnight and then transferred to 20% sucrose solution in 100 mmol/L PB at 4°C. After cryoprotection, the brain was rapidly frozen by heat exchange from vaporized carbon dioxide gas (−70°C) connected to a carbon dioxide gas tank. The frozen brain was kept in a cryostat until adjusting to the surrounding −20°C-temperature. Brain pieces, 12 µm thick, were cut with a cryostat and pasted onto glass slides. Brain slices were incubated with primary antibody, mouse monoclonal antibody to detect human phosphorylated tau and paired-helical filaments (AT8, Innogenetics, Gent, Belgium) overnight at 4°C, and next with biotinylated antibody to rabbit IgG (Vector Laboratories, Burlingame, CA, USA) for 1 h at room temperature. Then the sections were incubated with avidin peroxidase using a VECTASTAIN ABC kit (Vector Laboratories) for 1 h at room temperature. All sections were rinsed several times with PBS between incubations. Labeling was revealed by DAB, which yielded a dark brown color. After incubation, cellular nuclei were stained with Mayer's hematoxylin solution (WAKO, Osaka, Japan). Histological analysis of the lateral globus pallidus (bregma −1.60 mm, coronal section), auditory cortex, amygdala (bregma −2.18 mm, coronal section), and ventral hippocampus (bregma −3.64 mm, coronal section) was performed by fluorescence microscope BZ-9000 (Keyence, Osaka, Japan).

To evaluate the affected brain region regarding behavioral abnormalities with cognitive dysfunction, we assessed male tauopathy model mice at 6 months of age. Histological analysis of the cingulate cortex (bregma −0.10 mm, coronal section), anterior cortical amygdaloid nucleus and dorsal hippocampus (bregma −1.06 mm, coronal section) was performed in the same way as above.

### Immunoblotting

Tau protein levels of brains of mice at 6 months of age were determined by homogenizing brains (whole cortex and whole hippocampus of each mouse) in 2 ml/g tissue of ice-cold high-salt reassembly buffer (RAB-HS) (0.1 M MES, 1 mM EGTA, 0.5 mM MgSO4, 0.75 M NaCl, 0.02 M NaF, 1 mM PMSF, and protease inhibitor cocktail (Roche Diagnostics, Switzerland), followed by centrifugation at 50,000× g for 40 min at 4°C. Protein concentrations were determined and SDS-PAGE, followed by western blot analysis, was performed as described [63], [64].

### Image and data analysis

The applications used for the behavioral studies (Image LD, Image EP, Image PS, Image SI, Image YM, Image CSI, Image BM, Image FZ, and Image TS) were based on the public domain NIH Image program (developed at the U.S. National Institutes of Health available at http://rsb.info.nih.gov/nih-image/) and the ImageJ program (http://rsb.info.nih.gov/ij/), which were modified for each test by Miyakawa (available through O'Hara & Co.). Statistical analysis was conducted using StatView (SAS Institute, Cary, NC, USA). Normality was analyzed by Kolmogorov- Smirnov test, and the equality of distributions by Bartlett test. Data were analyzed by two-tailed t-test (Student's t-test, Welch's t-test), Mann-Whitney's U-test, one-way analysis of variance (ANOVA), or two-way mixed model ANOVA for each behavioral analysis. Significant differences were defined as *p*-value<0.05. Values in graphs were expressed as mean ± SEM.

## Supporting Information

Figure S1 **Time course of each task of behavioral analysis.** GC: General conditions, LD: Light/Dark transition test, OF: Open field test, EP: Elevated-plus maze, PS: Porsolt forced swim test, HP: Hot plate test, SI: Social interaction test, RR: Rotarod treadmill test, PPI: Prepulse inhibition test, YM: Y-maze test, CSI: Crawley's social interaction test, BM: Barnes maze test, MW: Morris water maze test, FZ: Fear conditioning test, TS: Tail suspension test.
(TIF)

Figure S2 **General conditions in 13-week-old mice.** Body weight (A), Rectal temperature (B), Grip strength (C), Wire hang duration (D). No statistical significance was observed at this age between tauopathy model mice and wild type mice. Controls: wild type mice (n = 19); Mutants: tauopathy model mice (n = 16). Tested with Student's *t*-test.
(TIF)

Figure S3 **Light/dark transition test.** Distance traveled (A), Stay time in the light chamber (B), Number of transitions between light and dark chambers (C), First latency to enter light chamber from dark chamber (D). No statistical significance was observed between tauopathy model mice and wild type mice. Controls: wild type mice (n = 19); Mutants: tauopathy model mice (n = 16). Tested with Student's t-test.
(TIF)

Figure S4 **Rotarod treadmill test (A) and hot plate test (B).** In rotarod treadmill test, tauopathy model mice tended to be hyperactive compared to wild type mice. However, no statistical significance was observed. Tested with two-way mixed model ANOVA, *F*(1, 33) = 2.526, *p* = 0.1215, between groups). In hot plate test, latency to react to stimulation was significantly reduced in tauopathy model mice. Controls: wild type mice (n = 19); Mutants: tauopathy model mice (n = 16). Tested with Student's *t*-test.
(TIF)

Figure S5 **Tail suspension test.** One wild type mouse dropped during the test and failed to complete the task. No statistical significance was observed between tauopathy model mice and wild type mice (*F*(1, 32) = 0.884, *p* = 0.3524, between groups). Controls: wild type mice (n = 18); Mutants: tauopathy model mice (n = 16). Tested with two-way mixed model ANOVA.
(TIF)

Figure S6 **One-chamber social interaction test.** Genotypic mismatch was found in one pair (tauopathy model pair) and they could not be analyzed. Total duration of contacts (A), total number of contacts (B), total duration of active contacts (C), mean duration per contact (D), total distance traveled during the test (E). No statistical significance was observed between tauopathy model mice and wild type mice. Controls: wild type mice (n = 9); Mutants: tauopathy model mice (n = 7). Tested with Student's *t*-test.
(TIF)

Figure S7 **Barnes circular maze test.** Training session (A–C), probe test 24 h after the last (24^th^) training (D). No statistical significances were observed in the training session (Distance to 1^st^: *F*(1, 33) = 0.458, *p* = 0.5031, between groups. Latency to 1^st^: *F*(1, 33) = 0.153, *p* = 0.6079, between groups. Error to 1^st^: *F*(1, 33) = 0.069, *p* = 0.7937), between groups. Tested by two-way mixed model ANOVA. In the probe test, time spent with the hole next to the target significantly differed between tauopathy model mice and wild type mice. No statistical significance was observed with the target. Controls: wild type mice (n = 19); Mutants: tauopathy model mice (n = 16). Tested with Student's *t*-test.
(TIF)
