# Supplementary figures and images for: P301S Mutant Human Tau Transgenic Mice Manifest Early Symptoms of Human Tauopathies with Dementia and Altered Sensorimotor Gating
Source: PLoS One. 2011 Jun 15;6(6):e21050. doi: 10.1371/journal.pone.0021050 (PMC3115982; doi:10.1371/journal.pone.0021050)

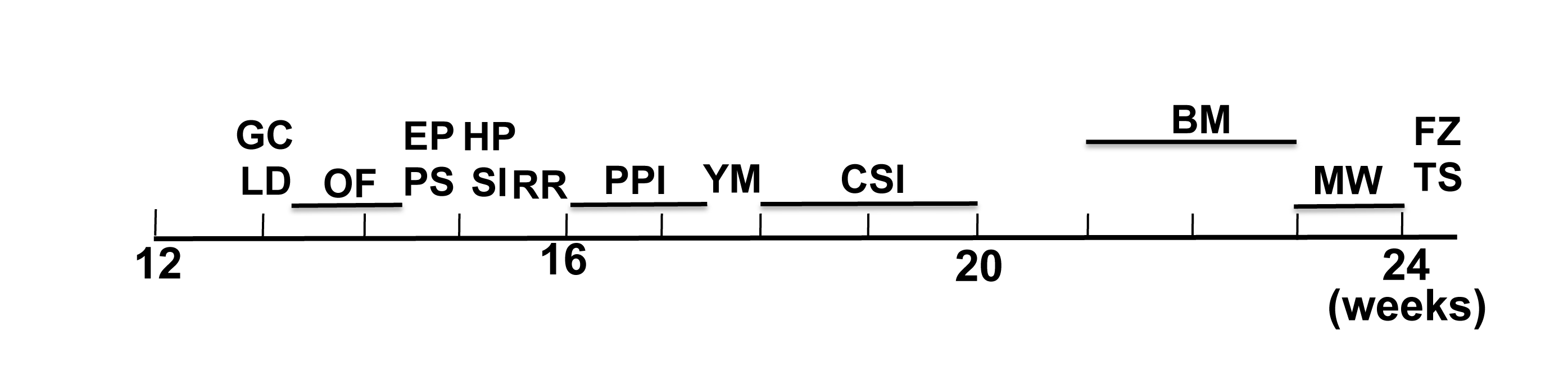

Supplement: Figure S1 — Time course of each task of behavioral analysis. GC: General conditions, LD: Light/Dark transition test, OF: Open field test, EP: Elevated-plus maze, PS: Porsolt forced swim test, HP: Hot plate test, SI: Social interaction test, RR: Rotarod treadmill test, PPI: Prepulse inhibition test, YM: Y-maze test, CSI: Crawley's social interaction test, BM: Barnes maze test, MW: Morris water maze test, FZ: Fear conditioning test, TS: Tail suspension test. (TIF) [file pone.0021050.s001.tif]

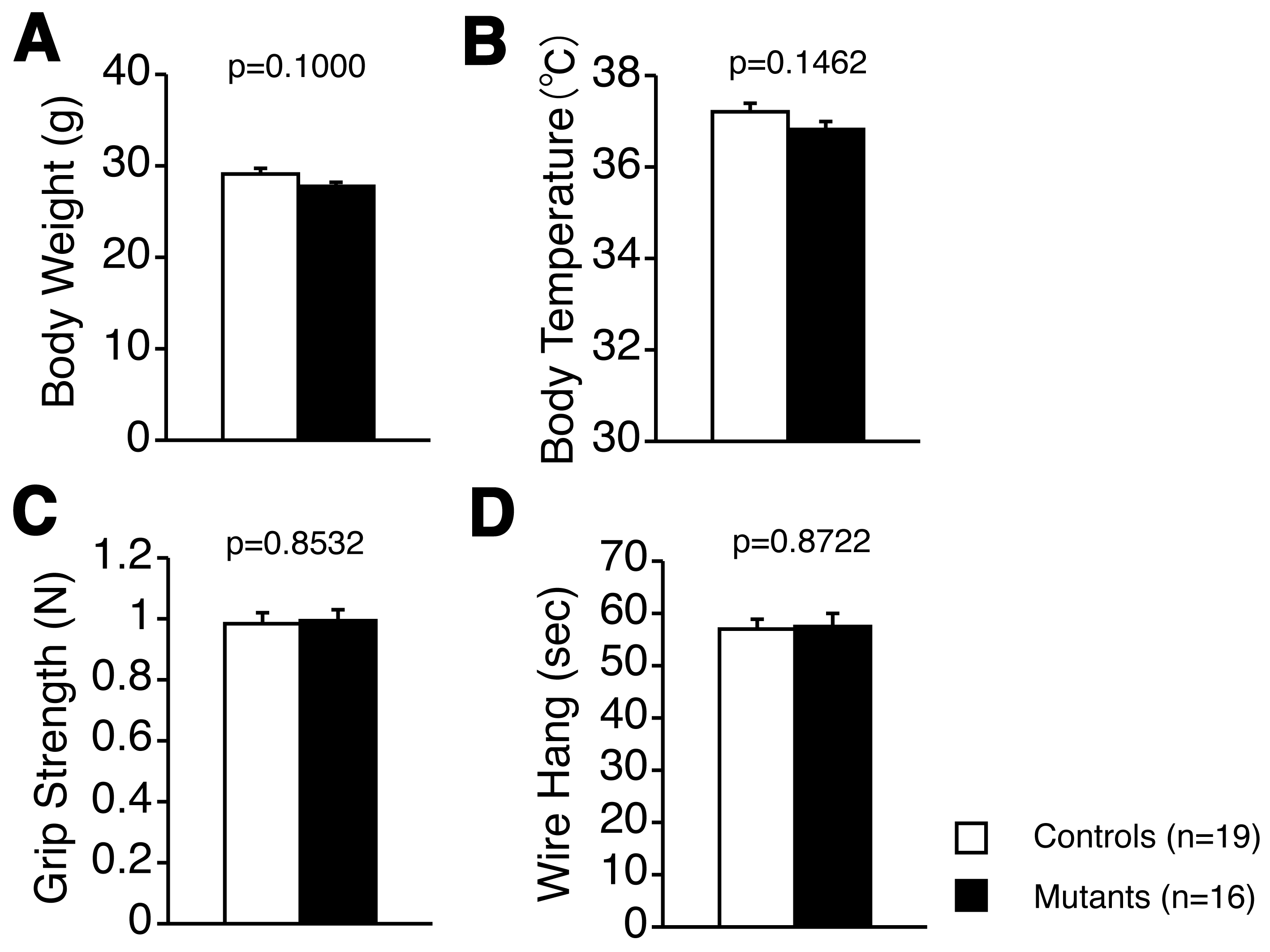

Supplement: Figure S2 — General conditions in 13-week-old mice. Body weight (A), Rectal temperature (B), Grip strength (C), Wire hang duration (D). No statistical significance was observed at this age between tauopathy model mice and wild type mice. Controls: wild type mice (n = 19); Mutants: tauopathy model mice (n = 16). Tested with Student's t-test. (TIF) [file pone.0021050.s002.tif]

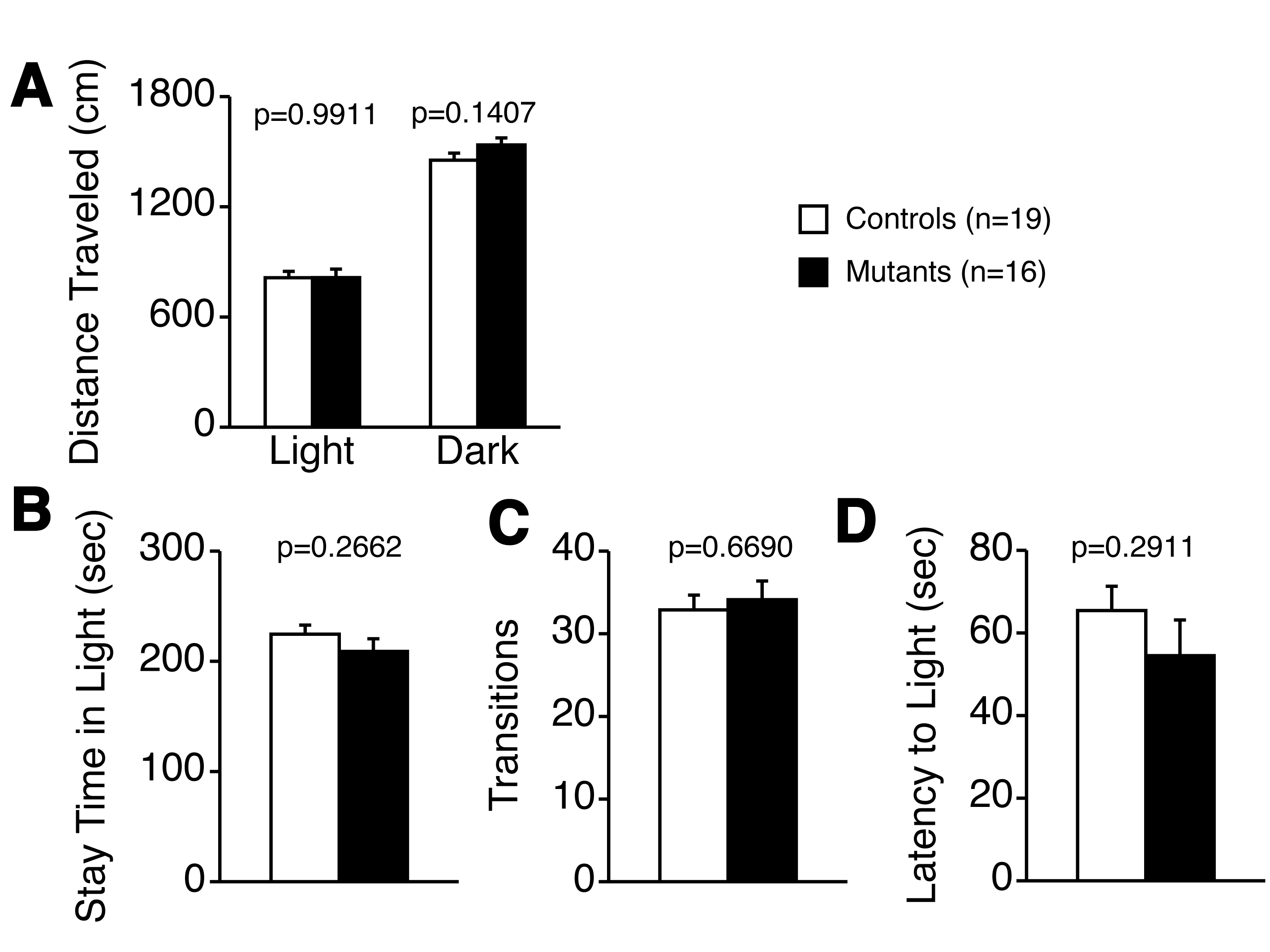

Supplement: Figure S3 — Light/dark transition test. Distance traveled (A), Stay time in the light chamber (B), Number of transitions between light and dark chambers (C), First latency to enter light chamber from dark chamber (D). No statistical significance was observed between tauopathy model mice and wild type mice. Controls: wild type mice (n = 19); Mutants: tauopathy model mice (n = 16). Tested with Student's t-test. (TIF) [file pone.0021050.s003.tif]

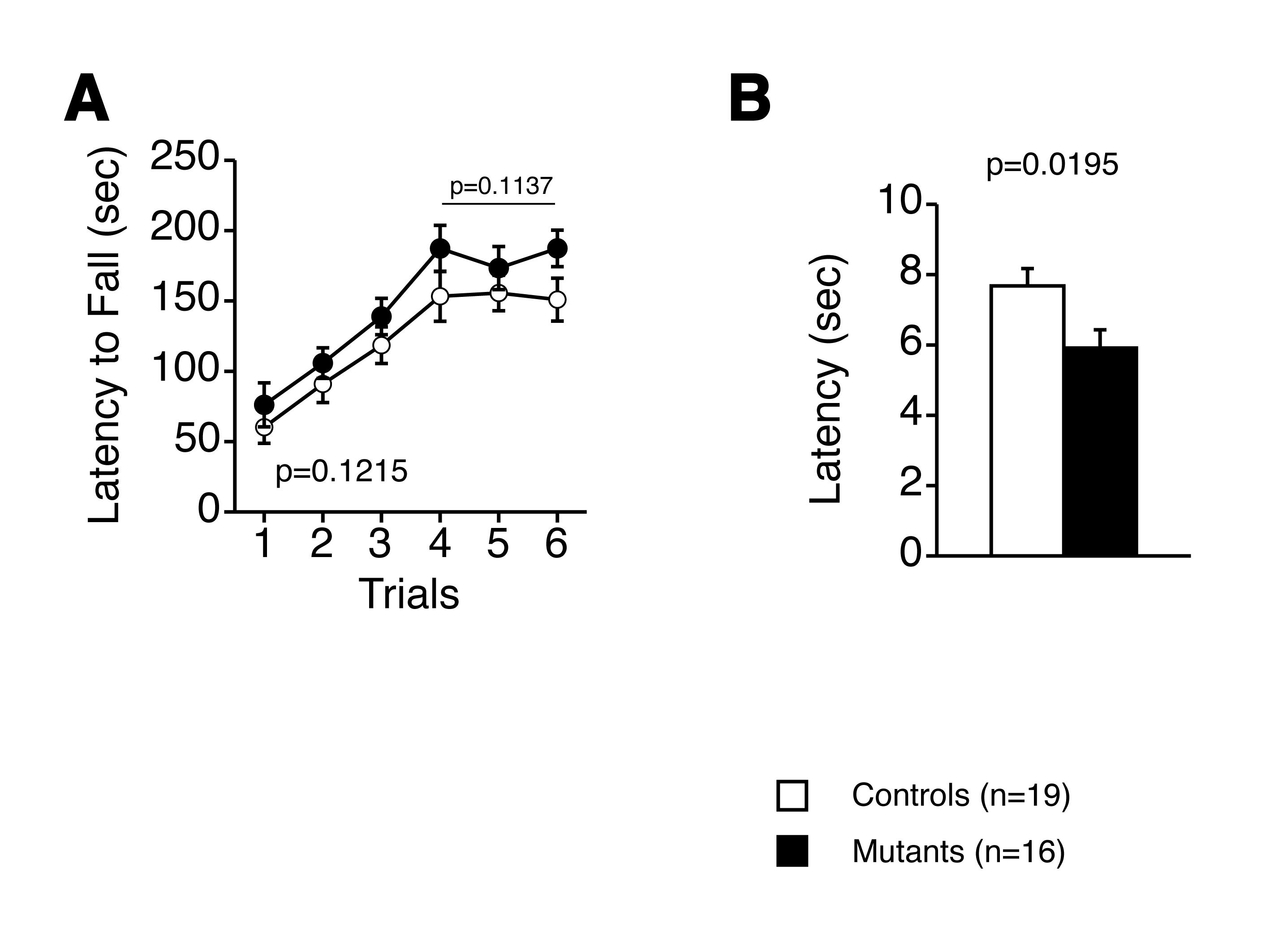

Supplement: Figure S4 — Rotarod treadmill test (A) and hot plate test (B). In rotarod treadmill test, tauopathy model mice tended to be hyperactive compared to wild type mice. However, no statistical significance was observed. Tested with two-way mixed model ANOVA, F(1, 33) = 2.526, p = 0.1215, between groups). In hot plate test, latency to react to stimulation was significantly reduced in tauopathy model mice. Controls: wild type mice (n = 19); Mutants: tauopathy model mice (n = 16). Tested with Student's t-test. (TIF) [file pone.0021050.s004.tif]

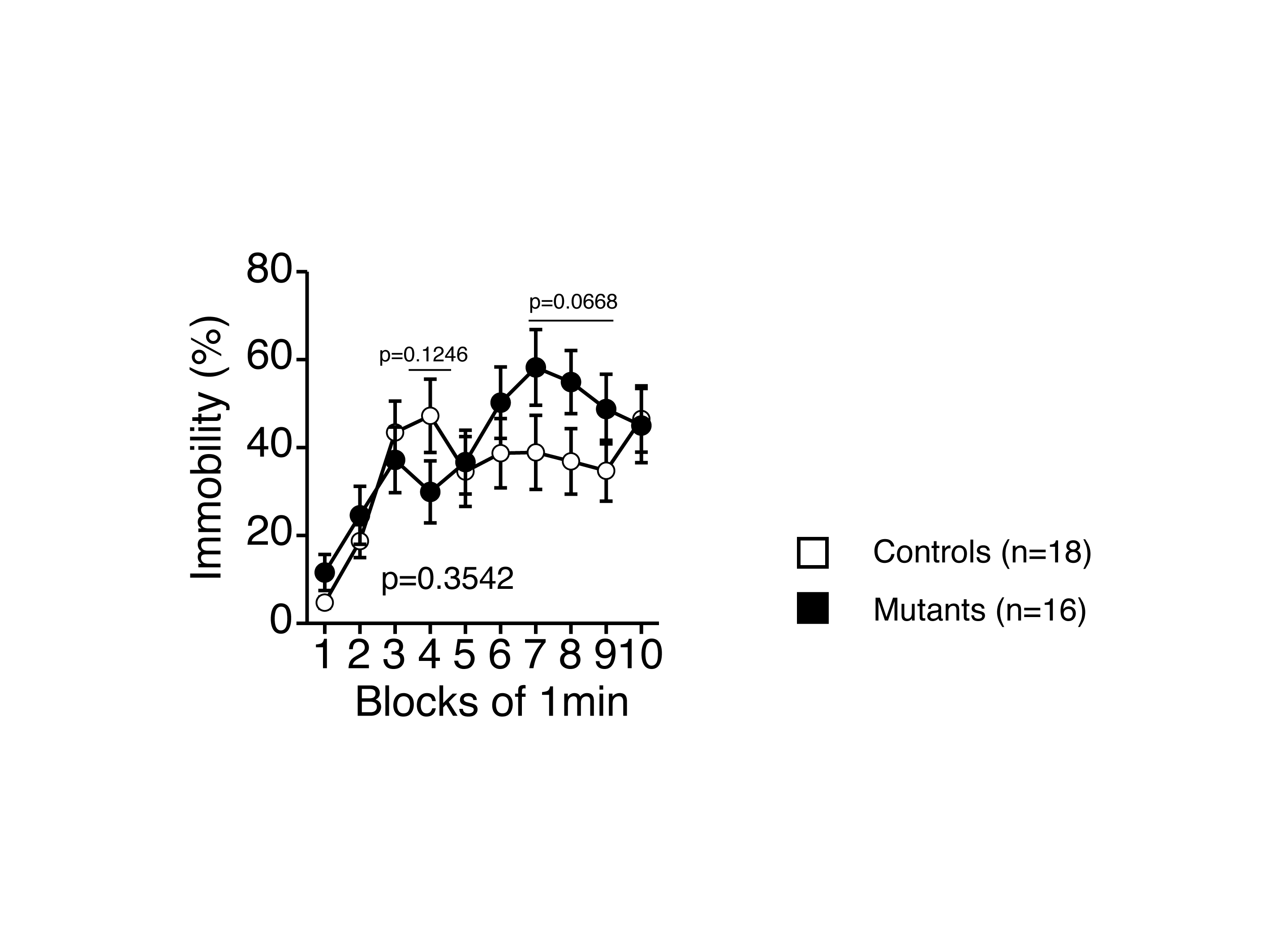

Supplement: Figure S5 — Tail suspension test. One wild type mouse dropped during the test and failed to complete the task. No statistical significance was observed between tauopathy model mice and wild type mice (F(1, 32) = 0.884, p = 0.3524, between groups). Controls: wild type mice (n = 18); Mutants: tauopathy model mice (n = 16). Tested with two-way mixed model ANOVA. (TIF) [file pone.0021050.s005.tif]

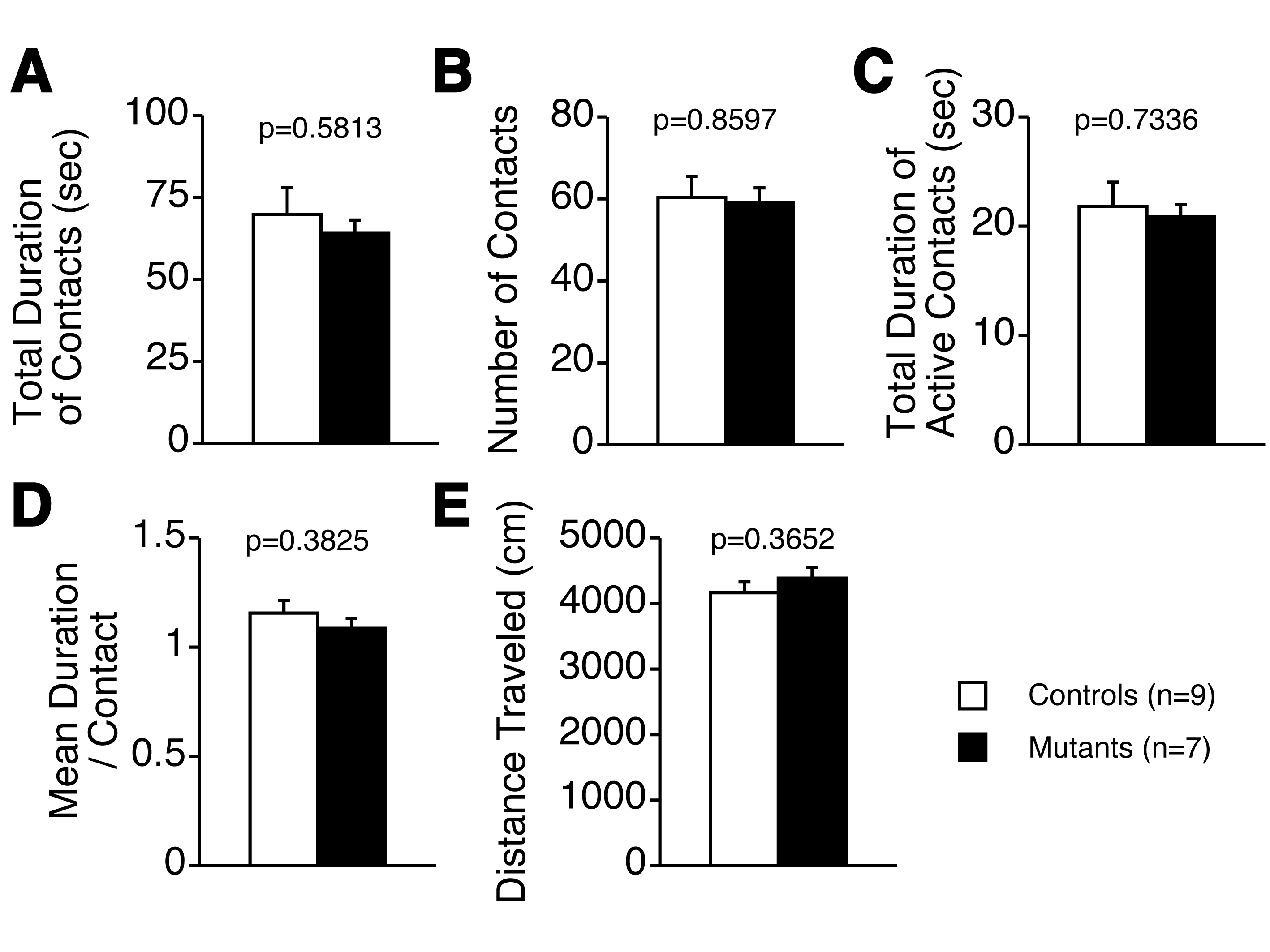

Supplement: Figure S6 — One-chamber social interaction test. Genotypic mismatch was found in one pair (tauopathy model pair) and they could not be analyzed. Total duration of contacts (A), total number of contacts (B), total duration of active contacts (C), mean duration per contact (D), total distance traveled during the test (E). No statistical significance was observed between tauopathy model mice and wild type mice. Controls: wild type mice (n = 9); Mutants: tauopathy model mice (n = 7). Tested with Student's t-test. (TIF) [file pone.0021050.s006.tif]

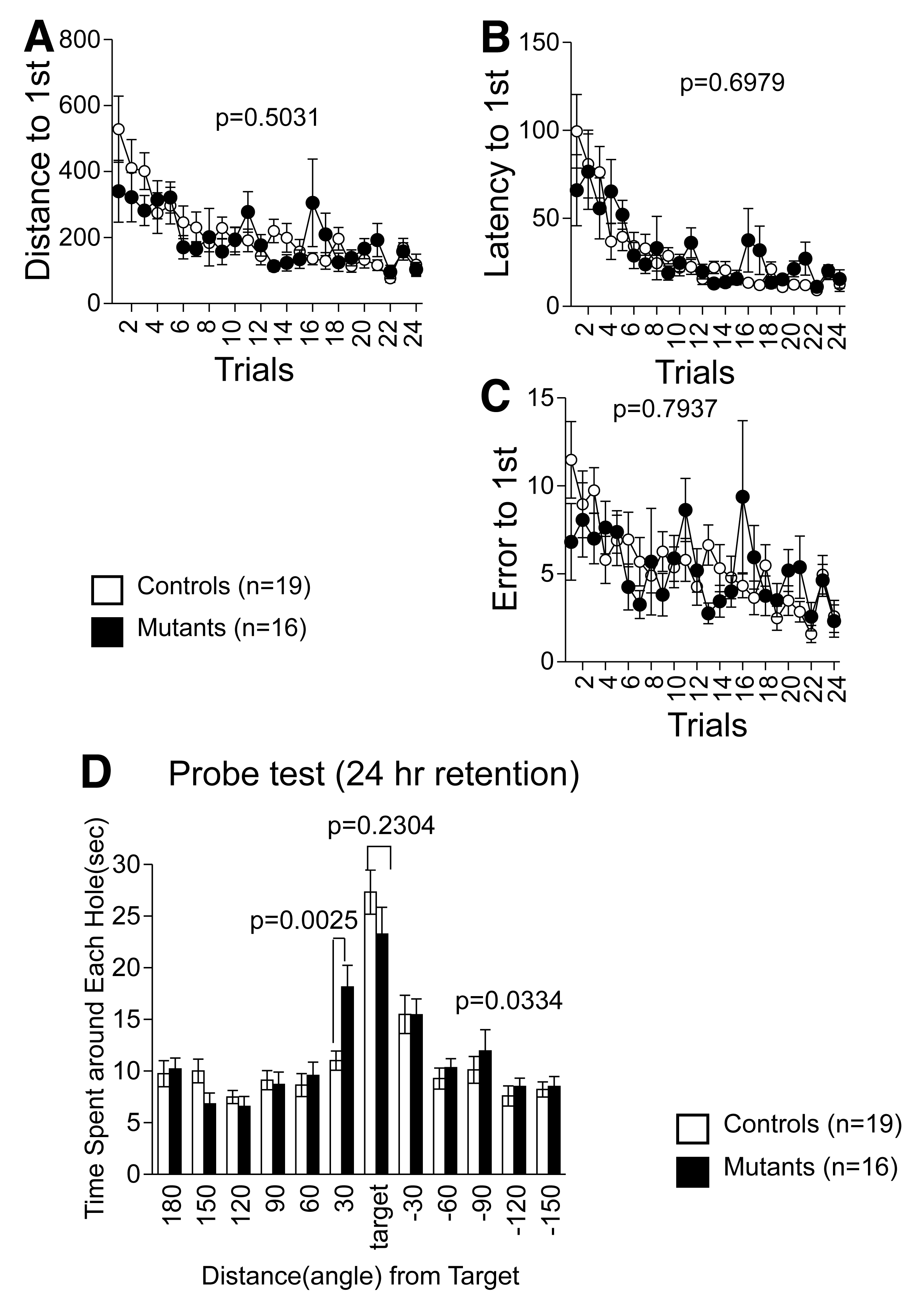

Supplement: Figure S7 — Barnes circular maze test. Training session (A–C), probe test 24 h after the last (24th) training (D). No statistical significances were observed in the training session (Distance to 1st: F(1, 33) = 0.458, p = 0.5031, between groups. Latency to 1st: F(1, 33) = 0.153, p = 0.6079, between groups. Error to 1st: F(1, 33) = 0.069, p = 0.7937), between groups. Tested by two-way mixed model ANOVA. In the probe test, time spent with the hole next to the target significantly differed between tauopathy model mice and wild type mice. No statistical significance was observed with the target. Controls: wild type mice (n = 19); Mutants: tauopathy model mice (n = 16). Tested with Student's t-test. (TIF) [file pone.0021050.s007.tif]
